# Lower limb biomechanics during running in individuals with Achilles tendinopathy: a systematic review

**DOI:** 10.1186/1757-1146-4-S1-O32

**Published:** 2011-05-20

**Authors:** Shannon E Munteanu, Christian J Barton

**Affiliations:** 1 Department of Podiatry, Faculty of Health Sciences, La Trobe University, Melbourne, Victoria, 3086, Australia; 2 Musculoskeletal Research Centre, Faculty of Health Sciences, La Trobe University, Melbourne, Victoria, 3086, Australia; 3 School of Physiotherapy, Faculty of Health Sciences, La Trobe University, Melbourne, Victoria, 3086, Australia

## Background

Achilles tendinopathy is common and its aetiology is considered multifactorial. Abnormal biomechanics of the lower limb has been speculated to be a risk factor for Achilles tendinopathy. The aim of this study was to systematically review the existing literature to identify, critique and summarise lower limb biomechanical factors associated with Achilles tendinopathy.

## Methods

We searched electronic bibliographic databases (Medline, EMBASE, Current contents, CINAHL and SPORTDiscus) in January 2010. All prospective cohort and retrospective case-control studies that evaluated biomechanical factors (temporospatial parameters, lower limb kinematics, dynamic plantar pressures, kinetics [ground reaction forces and joint moments] and muscle activity) associated with midsubstance Achilles tendinopathy were included. Methodological quality of included studies was evaluated using the Quality Index. To evaluate the magnitude of differences between cases (those with Achilles tendinopathy) and controls (those without Achilles tendinopathy), effect sizes (Cohen’s d) were calculated.

## Results

Nine studies were identified. Two studies were a prospective cohort study design and seven were case-control studies. The methodological quality of the identified studies was varied, with Quality Index scores ranging from 4 to 15 out of a possible score of 17. All studies analysed running biomechanics. Cases were found to have increased eversion range of motion of the rearfoot, reduced maximum leg abduction, reduced ankle joint dorsiflexion velocity, reduced knee flexion during gait, altered plantar pressures and ground reaction forces as well as reduced peak tibial external rotation moment. Further, cases displayed differences in the timing and amplitude of a number of lower limb muscles. Notably, the onset of tibialis anterior was delayed and the duration of soleus and lateral gastrocnemius was increased. In addition, cases displayed reductions in the amplitude of gluteus medius and rectus femoris.

## Conclusions

There are differences in lower limb biomechanics between those with and without Achilles tendinopathy. These findings have implications for the prevention and management of the condition. However, future well-designed studies are required to determine if interventions such as foot orthoses and/or physical therapy targeting identified differences in those with Achilles tendinopathy are effective at preventing and/or treating the condition.

